# Geographic Trends and Geospatial Analysis of Head and Neck Fellowship‐Trained Surgeons

**DOI:** 10.1002/hed.70016

**Published:** 2025-08-14

**Authors:** Jad Zeitouni, Harry May, Hannah Seo, Preston Thipaphay, Nosayaba Osazuwa‐Peters, Mark A. Varvares, Yusuf Dundar

**Affiliations:** ^1^ Massachusetts Eye and Ear Department of Otolaryngology‐Head and Neck Surgery Boston Massachusetts USA; ^2^ School of Medicine Texas Tech University Health Sciences Center Lubbock Texas USA; ^3^ Department of Head and Neck Surgery & Communication Sciences Duke University School of Medicine Durham North Carolina USA; ^4^ Department of Population Health Sciences Duke University School of Medicine Durham North Carolina USA; ^5^ Duke Cancer Institute Duke University Durham North Carolina USA; ^6^ Harvard Medical School Otolaryngology‐Head and Neck Surgery, Massachusetts Eye and Ear Boston Massachusetts USA

**Keywords:** access to care, geospatial analysis, head and neck cancer, head and neck fellowship, workforce

## Abstract

**Background:**

Disparities in access to otolaryngology and cancer care exist across the United States. However, little is known about the geographic distribution of fellowship‐trained head and neck cancer (HNC) surgeons.

**Methods:**

A cross‐sectional study of American Head and Neck Society (AHNS) fellowship graduates from July 1, 1997 to June 30, 2022 was conducted. Geospatial and statistical analysis was conducted to assess current practice location and correlations with training regions.

**Results:**

Among 688 graduates, 622 practice in the US or Canada. Most graduates remained in the region of their training. Geospatial analysis showed concentration of graduates in urban areas, with 152 of 3142 US counties having higher‐than‐expected density. Underserved regions were identified in the southeastern US, southern border, and western states.

**Conclusions:**

Head and neck surgical fellowship graduates predominantly practice in large urban areas, leaving rural and underserved regions with limited access to complex HNC care. Strategic interventions are needed to address these gaps.

## 1 Introduction

The need for otolaryngology care in North America has surged. There is approximately 1 otolaryngologist for every 27 778 individuals in the US [1]. Access to otolaryngology is significantly more limited for rural populations [1, 2, 3]. Moreover, 61.8% of otolaryngologists practice in counties with more than one million residents [3]. Only 7.2% of otolaryngologists practice in counties with less than 75 000 residents [3]. In the US population alone, the estimated shortfall of otolaryngologists in 2025 is over 11 000, up from 8600 in 2012 [4]. The number of otolaryngology residency positions has grown to address this need as residency positions in the US have risen 45% (from 264 spots to 382) over the past 18 years [5, 6]. Despite this significant growth, the specialty remains increasingly competitive, with only one training position going unmatched in 2024 [6]. An added caveat to this workforce challenge is the increase in sub‐specialization through fellowships. The number of accredited AHNS fellowships has increased from 4 programs in 1997 to 38 programs in 2022, reflecting the growing recognition of the importance of specialized training to treat HNC in the US [7]. From 2011 to 2019, the percentage of graduating residents in otolaryngology pursuing fellowships increased by 15.9%; with more than half (61.5%) in 2019 pursuing advanced training after graduation [8]. Notably, there has been an increase in the number of otolaryngology residents applying to head and neck fellowship, from approximately 9% in 2011 to approximately 21% in 2019 [8].

Despite the significant growth in fellowship positions over the past 25 years and differential access to HNC by geographic region, little is known about where fellowship graduates ultimately practice in the US and Canada. This information is relevant as the existing literature suggests that many subspecialties, including head and neck surgery, are concentrated in urban and academic centers [9], and rural patients face challenges in accessing subspecialty care as a result [10]. At least 1 in 5 patients with head and neck cancer resides in a rural area, highlighting the need for access to head and neck subspecialty care for rural populations [11].

In this study, we aim to evaluate the trends and geographic distribution of AHNS‐accredited head and neck cancer fellowship graduates in the United States and Canada over the past 25 years.

## 2 Methods

We performed a cross‐sectional analysis using publicly available data published by the AHNS on graduates of head and neck fellowships from accredited AHNS programs between 1997 and 2022 [12]. Graduates from 2002 were not included in the graduate list published by the AHNS; hence, they were excluded from this analysis, as were graduates of fellowship programs not accredited by the AHNS during the study period. The study did not require IRB approval, as we utilized publicly accessible data.

Based on the published AHNS fellowship graduate list, we performed an internet search of each graduate, obtained data on academic department, hospital, and private practice affiliations, and supplemented this with data from Doximity [13] and US News & World Report [14] as needed. Data included the location (city and state) of residency training (local vs. international) and fellowship program, current US‐based practice location (if currently active) and status (practicing vs. retired). Next, we determined geographic coordinates and regional designations for all Canadian and US‐based fellowship‐trained surgeons (see Table 1), and for current active surgeons, we determined practice type (academic institution vs. community practice vs. private practice). Community hospitals were classified as non‐academic tertiary care hospitals, which can provide complex surgical services. Private practice was defined as a privately owned clinic or surgical center where the surgeon was listed on the facility's website as a care provider. For surgeons listed on both the website of a private practice clinic and a community hospital, they were categorized as community hospital practitioners.

**TABLE 1:** Training and current practice setting of AHNS fellowship graduates from 1997 to 2022.

	No. of graduates (*N* = 688)
Residency region (%)	
West	86 (12.5%)
Northeast	146 (21.2%)
Midwest	134 (19.5%)
South	150 (21.8%)
Puerto Rico	4 (0.6%)
Canada	71 (10.3%)
International	62 (9.0%)
Unknown	35 (5.1%)
Fellowship region (%)	
West	77 (11.2%)
Northeast	189 (27.5%)
Midwest	153 (22.2%)
South	197 (28.6%)
Canada	72 (10.5%)
Current region (%)	
West	108 (15.7%)
Northeast	134 (19.5%)
Midwest	116 (16.9%)
South	207 (30.1%)
Puerto Rico	5 (0.7%)
Guam	2 (0.3%)
Canada	50 (7.3%)
International	55 (8.0%)
Unknown, deceased, not practicing	11 (1.6%)
Academic setting (%)	
Academic	383 (55.7%)
Community	180 (26.2%)
Private practice	92 (13.4%)
Retired, unknown, deceased, not practicing	33 (4.8%)

## 3 Statistical Analysis

We performed all statistical analyses using GraphPad Prism (version 9.5.1). Initially, the Kolmogorov–Smirnov test assessed whether numerical data met assumptions of normality. Depending on the data distribution, either Spearman's rank correlation or Pearson's correlation was applied to evaluate the relationship between continuous variables. A correlation coefficient (*r*) above 0.8 was interpreted as a strong positive correlation. Odd ratios with 95% confidence intervals were reported to examine the geographic variables (e.g., locations of residency and fellowship) associated with the current locations of practicing graduates. The geographic variables were categorized as the regions of the US (e.g., Northeast, Midwest, South, and West) according to the US Census Bureau (Table 1). To examine how graduates were geographically dispersed at the county‐level in the US, we calculated a ratio of the number of graduates in each county to the county's population. This allowed us to develop a “hot and cold” map to indicate counties that may have disproportionately more or less head and neck surgeons. A ratio of each county population to the US population was divided by a ratio of the number of graduates in each county to the total number of graduates; for example Jefferson County, Alabama had 0.0020 (0.2% of US population) and 0.0071 (4 graduates out of total 565 practicing graduates in the US), generating a ratio of 3.55 (0.0071/0.0020). For counties without a graduate, the ratio of their county population to the US population was used and adjusted to the scale as a negative value, as described in previous physician density studies [15, 16, 17]. A value of 1 indicated that the observed number of graduates aligned with what would be expected based on population size. In contrast, values exceeding 1 signified a higher‐than‐anticipated concentration of graduates. Values less than 1 indicated a lower‐than‐anticipated concentration of graduates. Ratios were then scaled and mapped to create a US county‐level visualization, allowing for a clear depiction of regions with disproportionately high or low concentrations of practicing head and neck surgeons.

## 4 Results

### 4.1 Fellowship and Current Practice Distribution

We identified a total of 688 head and neck fellowship graduates (Table 1). Slightly more fellows trained in the South than anywhere else (28.6%), followed by the Northeast (27.5%), 22.2% in the Midwest, 11.2% in the West, and 10.5% in Canada. Similarly, for current practice locations, 30.1% practiced in the South, 19.5% in the Northeast, 16.9% in the Midwest, 15.7% in the West, 8.0% internationally, 7.3% in Canada, 0.7% in Puerto Rico, and 0.3% in Guam. Eleven of the graduates either could not be identified, are not currently practicing, or are deceased. Over half of graduates (55.7%) were in academic medicine, 26.2% employed in community hospitals, and 13.4% in private practice.

### 4.2 Growth of Fellowship Positions Over Time and Migration Patterns

**FIGURE 1:**
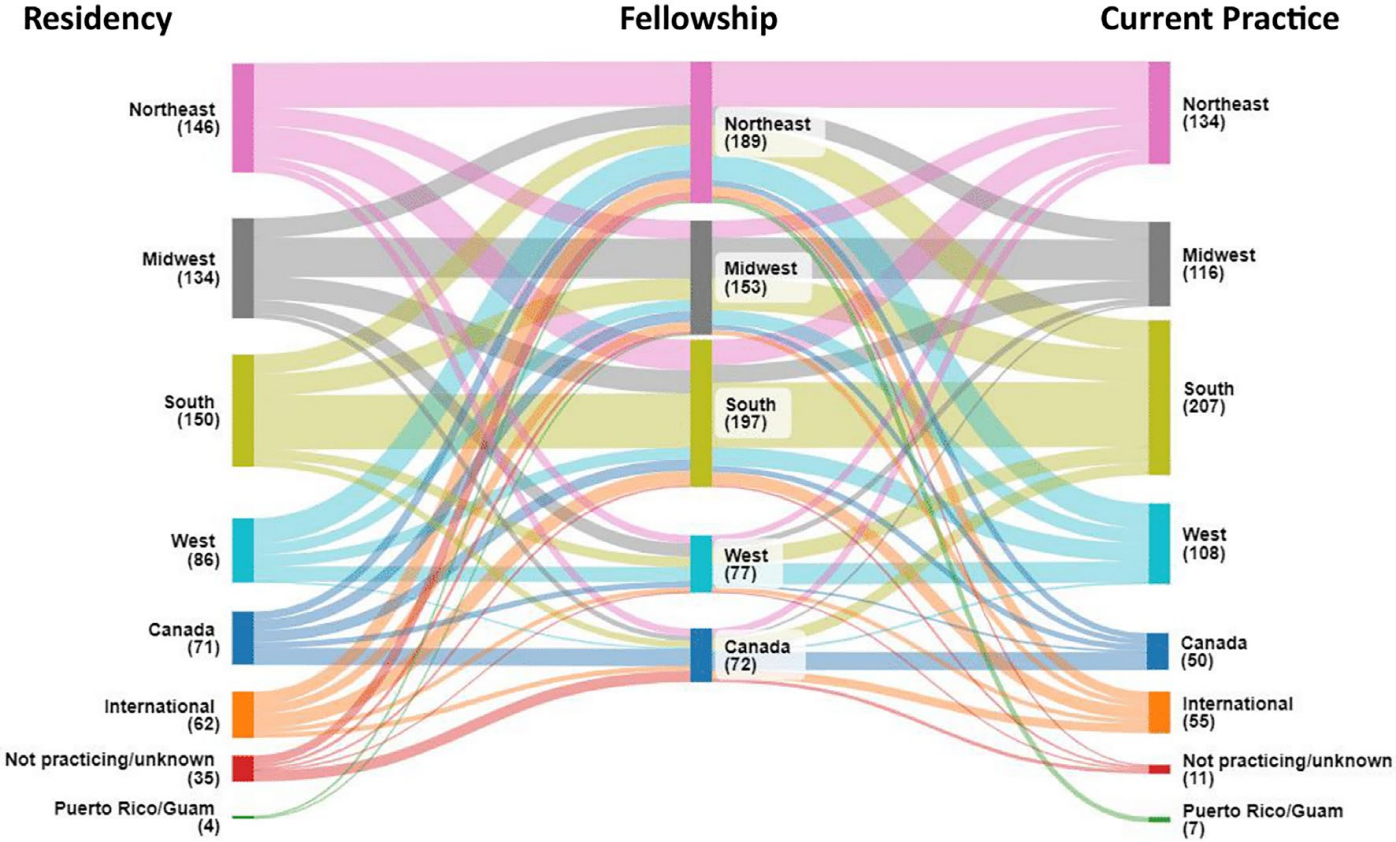
Sankey plot tracking graduates from residency regions to current practice regions. Thickness of the line indicates the number of graduates. The numbers in parentheses are the number of graduates. [Color figure can be viewed at wileyonlinelibrary.com]

**FIGURE 2:**
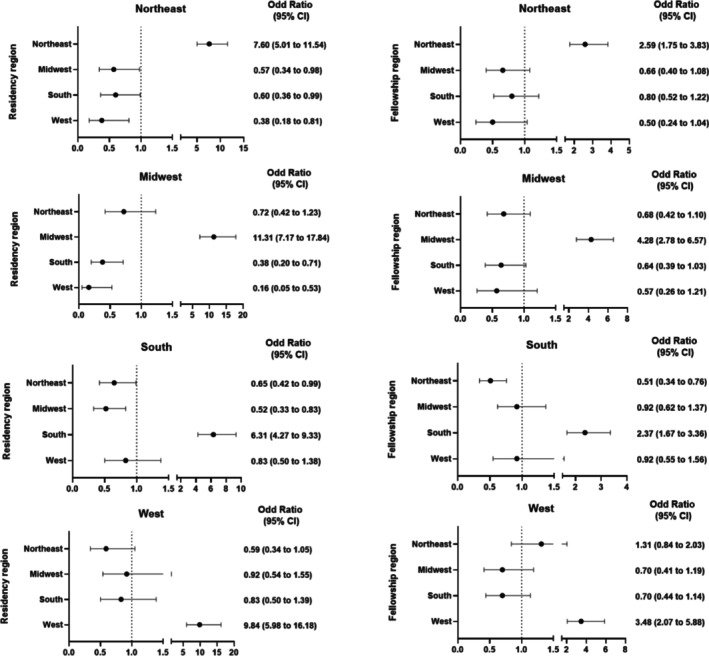
Odd ratio and 95% confidence interval for current practice region across graduates' residency and fellowship training region.

A Sankey plot (Figure 1) illustrated how graduates moved from residency regions to fellowship programs and ultimately to their current practice. Most regions retained their residency graduates for fellowship, with the exception of the West, where 40% of West‐trained residents pursued fellowships in the Northeast. However, graduates were more likely to practice in the region they trained for residency and fellowship (Figure 2).

### 4.3 Geographic Distribution

**FIGURE 3:**
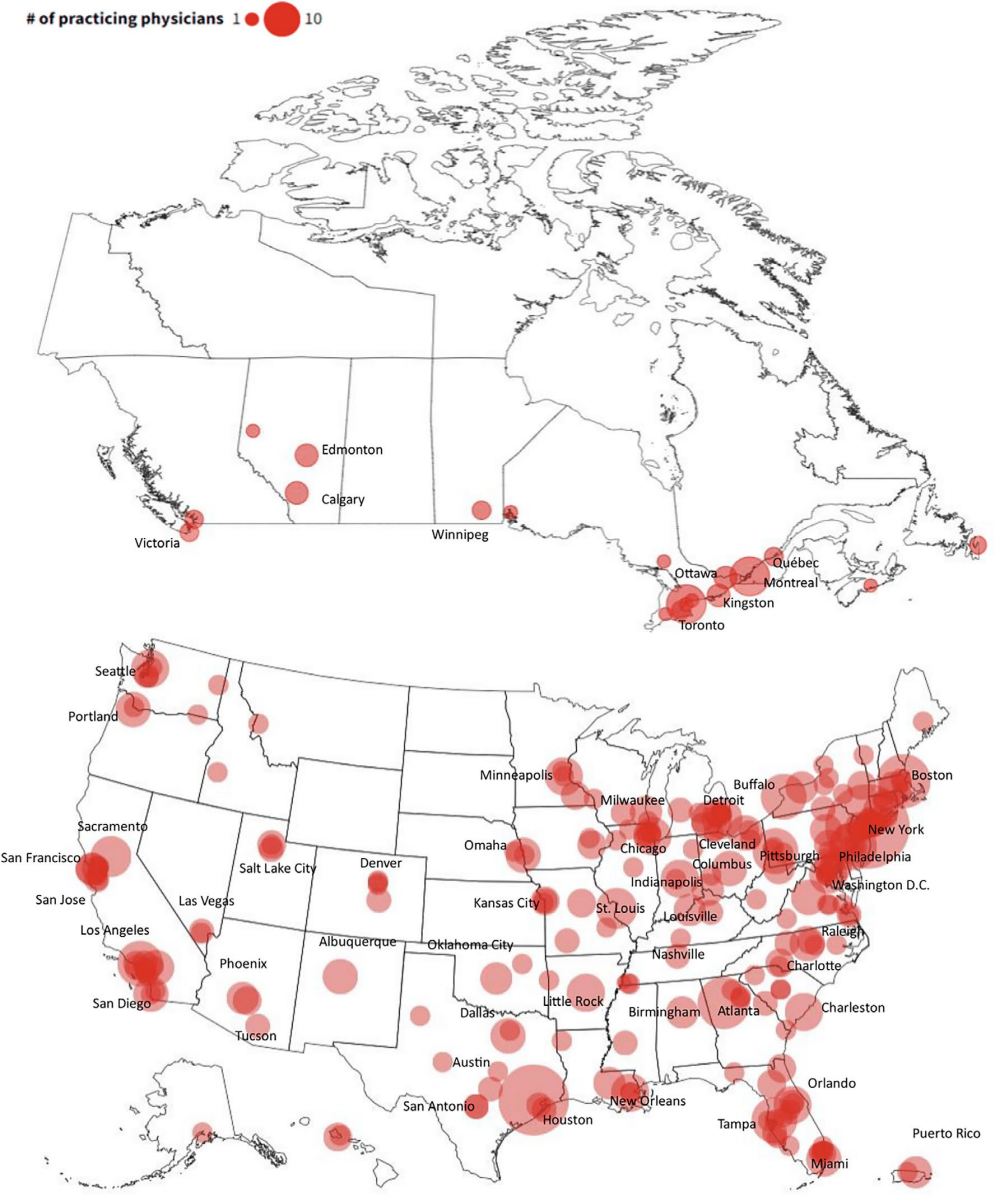
US and Canada Bubble map of current graduate location. Current locations of graduates are depicted as circles on the map. The size of circles represents the number of graduates. Major cities are labeled. International (*n* = 55) and Guam (*n* = 2) are not shown on the map. [Color figure can be viewed at wileyonlinelibrary.com]

Figure 3 illustrates the complete distribution of graduates, with 7 currently practicing in Guam or Puerto Rico and 55 practicing outside the US and Canada.

**FIGURE 4:**
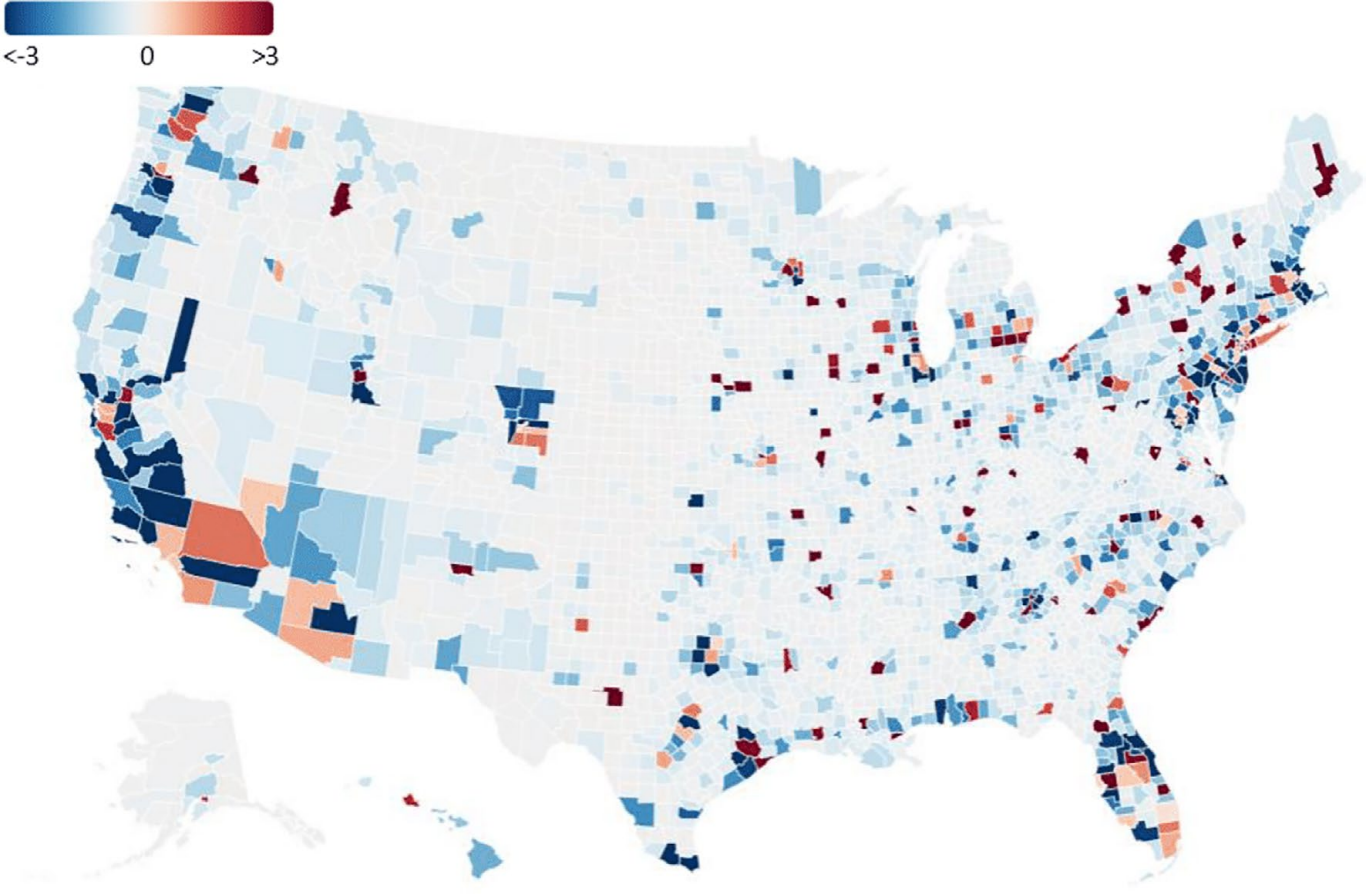
US hot‐cold density map of current graduates from 1997 to 2022. The number of current graduates per capita was calculated as the number of graduates (relative to the entire graduates in the US) of a given county divided by the population of the area (relative to the entire population in the US). A ratio close to −3 signified fewer graduates than expected based on a county population (blue), and 3 indicated more graduates than expected (red). [Color figure can be viewed at wileyonlinelibrary.com]

Figure 4 mapped the county‐level density of graduates, using a ratio of observed‐to‐expected numbers based on population. This “hot and cold” map shows which counties in the US have and do not have an adequate number of head and neck fellowship‐trained surgeons depicted via a color scheme of red and blue, with a darker shade indicating a higher (red) or lower (blue) ratio. A ratio of 1 indicates the expected number of surgeons for a county given its population.

Counties not shaded (gray) illustrate areas with the expected number of head and neck fellowship‐trained surgeons. The vast majority of these counties are rural counties with a small population size that would not indicate a need for a head and neck fellowship‐trained surgeon. Areas that are red demonstrate counties that have a higher‐than‐expected number of head and neck fellowship‐trained surgeons. Many of these counties are large urban areas with multiple academic centers and encompass cities such as Houston, Boston, and New York City. Finally, blue areas show counties that have a lower‐than‐expected number of head and neck fellowship‐trained surgeons. These counties range from rural counties (St. Louis, Minnesota) to suburban (Norfolk, Massachusetts) to urban counties (such as El Paso, Texas). Although many counties surrounding major cities had a lower‐than‐expected ratio, these counties do not necessarily struggle with access to HNC care, given their relative proximity to hubs with HNC care. Montour County, Pennsylvania, had the highest ratio; whereas Riverside County, California, had the lowest (Figure 4). New York contained the greatest number of counties (*n* = 14) exceeding a ratio of 1, followed by Florida (*n* = 10), Virginia (*n* = 8), Pennsylvania (*n* = 8), Texas (*n* = 7), and Ohio (*n* = 7).

### 4.4 Association Between Training Location and Current Practice

Figure 2 showed associations between fellowship region and the current practice region of graduates. Figure 2 also shows the association between the region fellowship graduates trained for residency and the current practice region, given the high proportion of residents practicing where they trained [18]. In general, residency and fellowship training from a region increased the odds of practicing in the same region. Graduates who completed residency (OR, 7.50; 95% CI, 4.96–11.33) and fellowship (OR, 2.71; 95% CI, 1.84–3.99) from the Northeast were more likely to practice in the Northeast (Figure 2). Similarly, completing residency (OR, 10.98; 95% CI, 7.03–17.15) and fellowship (OR, 4.34; 95% CI, 2.85–6.60) from the Midwest increased the odds of currently practicing in the Midwest (Figure 2). Also, residency (OR, 6.45; 95% CI, 4.38–9.50) and fellowship training (OR, 2.44; 95% CI, 1.73–3.44) in the South increased the odds of currently practicing in the South (Figure 2). Similarly, completing residency (OR, 9.40; 95% CI, 5.74–15.37) and fellowship (OR, 3.54; 95% CI, 2.12–5.92) from the West were associated with an increased odds of currently practicing in the West (Figure 2).

## 5 Discussion

There will be nearly 60 000 new cases of oral cavity and pharyngeal cancer diagnosed in the US in 2025, excluding cutaneous, thyroid, or other malignancies of the head and neck [19]. Most of these cases will be treated by a multidisciplinary team that includes a head and neck surgeon. The management of HNC is inherently complex, particularly for advanced disease, often requiring a multimodal approach including surgery, radiation therapy, and chemotherapy [20].

Access to advanced head and neck surgery may be determined, in part, by a patient's geographic location. We described geographic trends and distribution of head and neck graduates from fellowships accredited by AHNS. We found that fellowships grew consistently between 1997 and 2022, which is consistent with both the increasing interest among otolaryngology residents in sub‐specialization and the increased complexity in care for patients with HNC [8, 20]. It is worth noting that the number of AHNS‐accredited fellowships has increased to 50 programs since the end of this study period, as more existing unaccredited fellowships have become accredited by the AHNS. We also evaluated migration patterns among graduates, demonstrating that most fellows stay to train in the region where they did residency. Many fellowship graduates join academic institutions, but a number of graduates work outside of academics in community hospitals and private clinics. Graduates tend to work in and around major metropolitan areas, with few working in more rural areas in the country. Our geographic mapping illustrates the variations in access to HNC care across North America. This is consistent with previous literature that showed that specialized care in general is concentrated in academic and tertiary care centers [9]. Moreover, rural communities in the US have been shown to have worse HNC treatment outcomes; patients are more likely to be diagnosed at a later‐stage [21, 22, 23]. A recent study, however, did not find a difference in HNC outcomes in rural populations in Canada [24].

Although there have been two previous studies on the geographic distribution of head and neck fellowship graduates [25, 26], the current analysis adds to the literature by encompassing the complete list of graduates publicly published by the AHNS and examining the graduate distribution by county and city, providing a more granular picture of the geographic distribution of a primary workforce critical to the care of patients impacted by HNC, one of the few cancers in the US that has continued to increase in incidence [27]. The two previous studies looked at the concentration of graduates by state, which does not reveal distribution within individual states. Understanding the distribution of surgeons within individual states is particularly important in larger states with substantial rural or underserved populations. Beyond increased granularity, our study includes a significantly larger share of graduates. The two previous studies on this topic included AHNS graduates from 1997 to 2017 and from 2011 to 2020, respectively [25, 26]. Including the entire list of graduates provided by the AHNS from 1997 to 2022 provides a more complete and up‐to‐date picture of the distribution of fellowship‐trained head and neck surgeons.

Beyond the apparent rural–urban divide in access to HNC care, we identified the county‐level proportion and distribution of graduates relative to the county population, which is critical to the accessibility of HNC care [28]. Out of the 3142 counties in the US [29], 152 had ratios above 1, illustrating that access to HNC care is concentrated in less than 5% of counties. Large urban counties often exceed the expected ratio of fellowship‐trained surgeons relative to their population size, whereas many adjacent counties surrounding these urban areas fall below their expected number of surgeons. This is seen in large urban cities such as Houston, New Orleans, and Los Angeles. Individuals who reside in these cities or surrounding counties may not have to travel long distances to be able to access HNC care, compared to residents from rural counties. Patients with cancer living more than 50 miles from a specialty hospital have, on average, a poorer prognosis later‐stage diagnosis [30]. However, socioeconomically disadvantaged populations in these surrounding counties may still face transportation issues that make accessing care difficult [31, 32, 33].

We also identified a dearth of HNC surgeons on the US southern border, with no practicing surgeons across the entire Texas and New Mexico border, including El Paso, a major urban center. This area is home to a significant Latino population. Although there is recent literature on HNC incidence in some counties on the southern border [28], there are no studies comprehensively examining HNC outcomes on the southern border. There is, however, literature on other cancer disparities on the southern border, as studies have identified colorectal and cervical cancer outcome disparities among Latinos living near the southern border [34, 35]. With the nearest HNC surgeon hundreds of miles away, patients with HNC may face late diagnoses and delayed treatment initiation, both of which have a significant impact on survival [36, 37]. The southeast is another area that shows widespread areas without a HNC surgeon. States such as Alabama and Mississippi have only one county with a higher‐than‐expected proportion of HNC surgeons. This inevitably affects access to care and outcomes. A study in Alabama found widespread disparities related to the diagnosis of HNC in the state, with later‐stage diagnosis more prevalent in rural and black patients [38]. Availability of HNC surgeons might play a role in these disparities.

Other studies have evaluated the distribution of the otolaryngology workforce in the US and have consistently reported similar findings regarding distribution and access to care [1, 2, 39]. Specifically, rural areas tend to have lower distribution of otolaryngologists; the west coast and eastern half of the US (east of the Mississippi River) have high or adequate access to otolaryngologic care, and the center of the country (from west Texas to North Dakota) and western states have low access to otolaryngologic care [1, 2, 39].

Importantly, our results indicated an association between region of training and eventual practice, especially the regional location of residency training compared to the location of fellowship training. This is consistent with previously published data from the Association of American Medical Colleges (AAMC) found 54.6% of residents in general, and 42% of otolaryngology residents who completed residency between 2009 and 2018, remained to practice in the state they trained [18]. These trends highlight a potential solution to address the disproportionate distribution of HNC surgeons—increasing fellowship and residency training positions in areas with a clear need for HNC care. This strategy has been well documented in the literature [40].

The asymmetry of practice location shows that equitable access to HNC care does not only rely on increasing the number of fellowship positions but also on finding ways to incentivize graduates to practice in underserved areas of the country. An increased focus on recruiting HNC trainees from underserved areas is an important step in addressing this issue, as these trainees are more likely to practice in areas of physician shortage [41]. For example, growing up in a rural community is a strong factor for a healthcare provider to practice in a rural setting [41, 42]. Moreover, providing trainees more exposure in underserved or rural settings may also be an effective strategy to encourage trainees to practice in these communities [41]. Financial incentives and student loan repayment are also influential factors for providers to practice in a rural setting [43]. International medical graduates (IMGs) could also help provide coverage in underserved areas [41]. Finally, increasing job opportunities and placements in areas without an academic institution but with an existing tertiary care facility that is capable of supporting complex head and neck surgery may help address disproportionate workforce distribution and combat geographic gaps in head and neck cancer access to care.

## 6 Limitations

There are multiple limitations to this study. First, the study relies exclusively on the list of graduates from AHNS‐accredited fellowships between 1997 and 2022. Our data set does not comprehensively represent all head and neck cancer surgeons; it excludes graduates from 2002. Additionally, our data also excludes surgeons who were trained in a fellowship that was either non‐accredited or accredited by an organization other than the AHNS. This limited our study as multiple fellowships were not accredited by the AHNS until recently. Moreover, this growth of accredited fellowships does not reflect an equivalent growth of newly practicing head and neck surgeons as many of the newly accredited fellowships previously existed as unaccredited fellowships and were training fellows during that time. Although our study covers 24 years of graduates, there are graduates who completed fellowship prior to 1997 or after 2022 who are not included in our analysis. This study also relied exclusively on publicly available sources, which may include some omissions regarding current practice status. Additionally, this study did not examine other factors influencing practice location and limits our ability to fully explain regional distribution patterns.

Notwithstanding these limitations, this study offers a comprehensive overview of the geographic patterns and distribution of head and neck surgeons across North America. Future research should investigate how these differences in access affect diagnosis stage, time to treatment initiation, and patient outcomes in the identified regions.

## 7 Conclusion

Although the number of head and neck surgical fellowship positions has increased steadily over the last 25 years, graduates predominantly practice in large urban areas, leaving expansive rural and underserved regions as areas of head and neck oncology workforce shortage. Our findings highlight the need for an intentional and incentivized approach to attract trainees to these underserved areas. Future research should focus on real‐world HNC implications of geographic differences in provider distribution.

## Data Availability

Research data are not shared.
